# Supplementary figures and images for: Complete Genomic Sequence of Xanthomonas oryzae pv. oryzae Strain, LA20, for Studying Resurgence of Rice Bacterial Blight in the Yangtze River Region, China
Source: Int J Mol Sci. 2023 May 1;24(9):8132. doi: 10.3390/ijms24098132 (PMC10179132; doi:10.3390/ijms24098132)

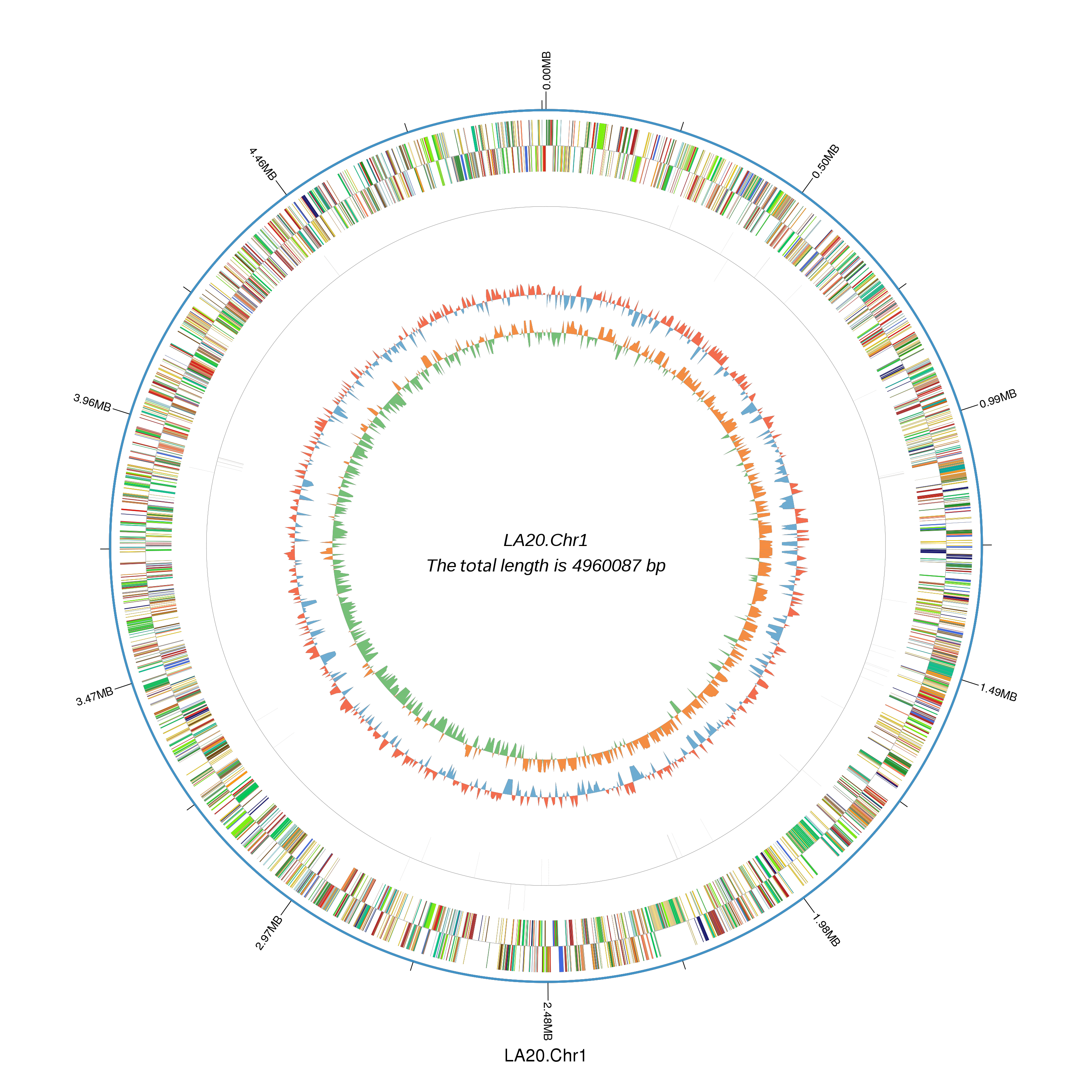

Supplement: Supplementary file 1 [file ijms-24-08132-s001.zip › Figure S1.tif]
